# Supplementary material for: Identification of long non-coding RNAs in advanced prostate cancer associated with androgen receptor splicing factors
Source: Commun Biol. 2020 Jul 23;3:393. doi: 10.1038/s42003-020-01120-y (PMC7378231; doi:10.1038/s42003-020-01120-y)
Supplement: Supplementary file 1 — Supplementary Information [file 42003_2020_1120_MOESM1_ESM.pdf]

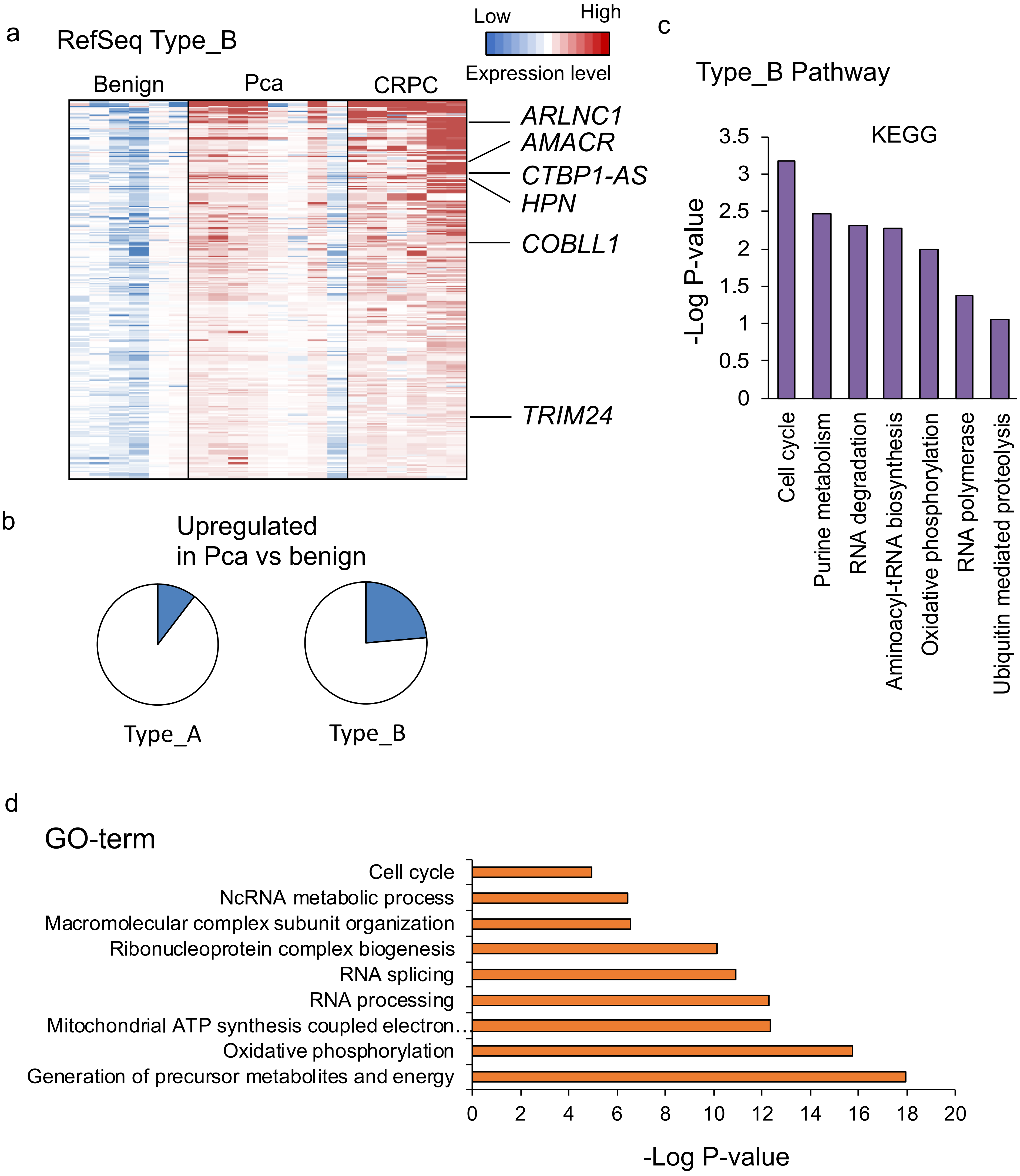

Supplementary Figure 1. Another type of genes highly expressed in CRPC tissues.
(a) Identification of additional genes involved in CRPC development. Expression level relative to benign prostate tissue is visualized as heatmap. RefSeq genes of Type_B, which are upregulated between CRPC and benign prostate significantly ($P < 0.05$, Mann-Whitney test). Some genes, which were previously reported to be associated with prostate cancer progression, are indicated.
(b) The rate of genes among Type_A and B whose expression levels are elevated in prostate cancer (Pca) compared with benign prostate tissues significantly ($P < 0.05$, Mann-Whitney test). We found that genes upregulated in Pca compared with benign prostate tissues are more enriched in Type_B rather than Tyepe_A ($P < 0.0001$, Chi-square test).
(c) KEGG pathway analysis of Type_B protein coding genes.
(d) Gene Ontology (GO)-term analysis of Type_B protein coding genes.

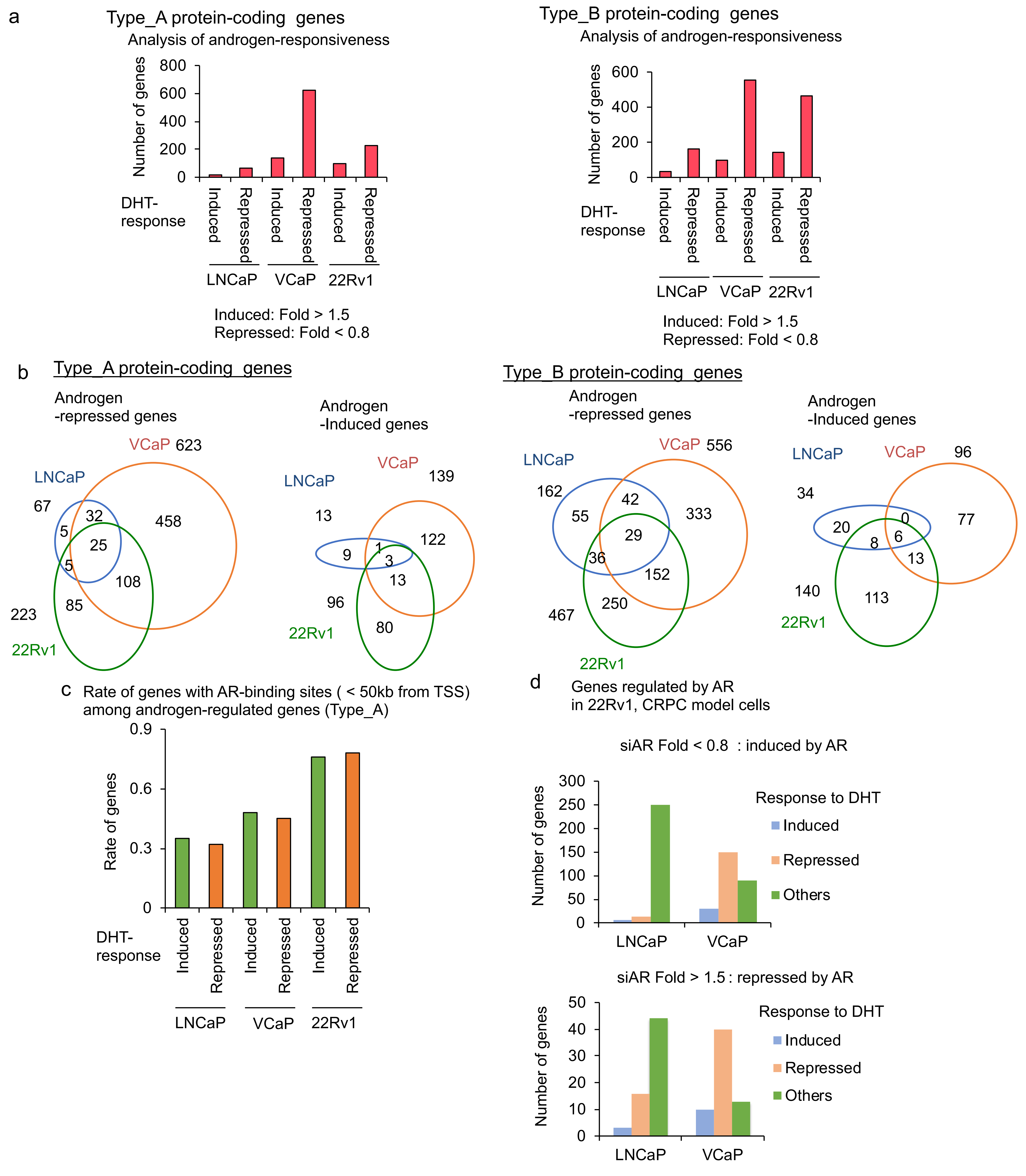

Supplementary Figure 2. Regulation of upregulated genes in CRPC tissues by androgen in prostate cancer cells.
(a) The numbers of genes upregulated in CRPC tissues and regulated by androgen in prostate cancer cells. We used RNA-seq data to examine each gene of Type_A and Type_B is induced (Fold > 1.5) or repressed (Fold < 0.8) by DHT (10 nM) treatment in LNCaP, VCaP, and 22Rv1 cells.
(b) Venn diagrams showed the overlap of repressed or induced genes in three prostate cancer cell lines.
(c) The rate of AR bindings among androgen-regulated genes (Type_A) identified in (a). TSS: transcription stat site
(d) AR-regulation of CRPC-associated genes in 22Rv1 cells is cell line specific. AR-regulated Type_A or Type_B genes in 22Rv1 cells, which are regulated by AR negatively (Fold > 1.5 by siAR) or positively (Fold < 0.8 by siAR), were used. Then we analyzed how many genes are regulated by DHT in LNCaP and VCaP cells. Androgen-induced (Fold > 1.5) or androgen-repressed (Fold < 0.8) genes were counted.

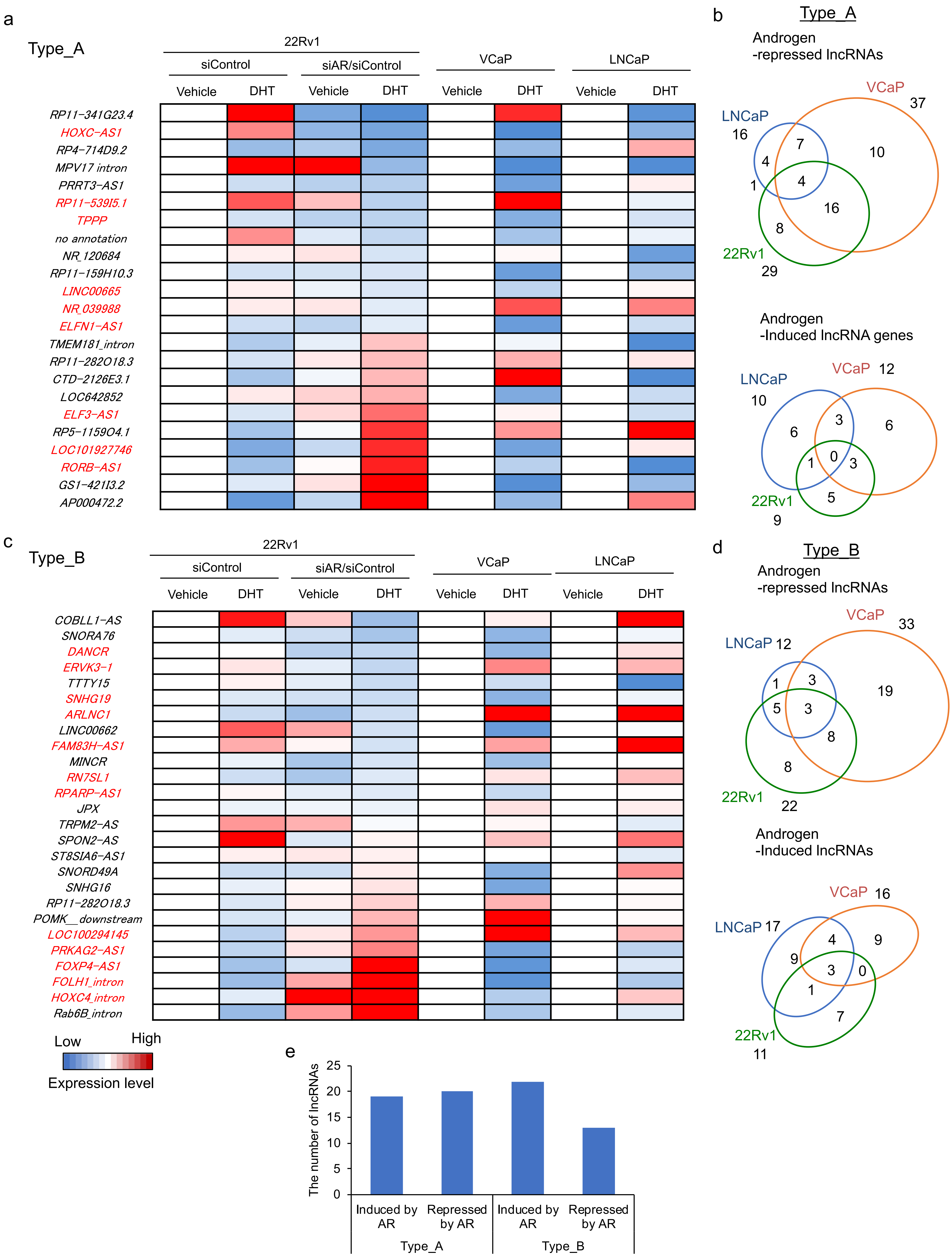

a
Type_A
22Rv1
siControl
siAR/siControl
VCaP
LNCaP
Vehicle
DHT
Vehicle
DHT
Vehicle
DHT
Vehicle
DHT
RP11−341G23.4
HOXC−AS1
RP4−714D9.2
MPV17 intron
PRRT3−AS1
RP11−539I5.1
TPPP
no annotation
NR_120684
RP11−159H10.3
LINC00665
NR_039988
ELFN1−AS1
TMEM181_intron
RP11−282O18.3
CTD−2126E3.1
LOC642852
ELF3−AS1
RP5−1159O4.1
LOC101927746
RORB−AS1
GS1−421I3.2
AP000472.2
b
Type_A
Androgen
-repressed lncRNAs
VCaP 37
LNCaP 16
22Rv1 29
7
4
10
4
1
16
8
Androgen
-Induced lncRNA genes
LNCaP 10
VCaP 12
22Rv1 9
3
6
6
1
0
3
5
c
Type_B
22Rv1
siControl
siAR/siControl
VCaP
LNCaP
Vehicle
DHT
Vehicle
DHT
Vehicle
DHT
Vehicle
DHT
COBLL1−AS
SNORA76
DANCR
ERVK3−1
TTTY15
SNHG19
ARLNC1
LINC00662
FAM83H−AS1
MINCR
RN7SL1
RPARP−AS1
JPX
TRPM2−AS
SPON2−AS
ST8SIA6−AS1
SNORD49A
SNHG16
RP11−282O18.3
POMK__downstream
LOC100294145
PRKAG2−AS1
FOXP4−AS1
FOLH1_intron
HOXC4_intron
Rab6B_intron
Low
High
Expression level
d
Type_B
Androgen
-repressed lncRNAs
VCaP 33
LNCaP 12
22Rv1 22
1
3
19
5
3
8
8
Androgen
-Induced lncRNAs
VCaP 16
LNCaP 17
22Rv1 11
4
9
9
3
0
1
7
e
The number of lncRNAs
25
20
15
10
5
0
Induced by AR
Repressed by AR
Induced by AR
Repressed by AR
Type_A
Type_B

Supplementary Figure 3. Regulation of AR-regulated lncRNAs overexpressed in CRPC tissues.
(a, c) Androgen or AR-mediated regulation of lncRNAs overexpressed in CRPC tissues (Type_A and Type_B) is shown by heatmap. Fold changes relative to siControl or vehicle control are shown. RNA-seq data in prostate cancer cells were used for this analysis. 21 lncRNAs examined in qPCR analysis (Figure 4a) are highlighted by red letters. (b, d) Venn diagrams showed the overlap of androgen-regulated lncRNAs. We identified androgen-repressed (Fold < 0.8) or androgen-induced (Fold > 1.5) lncRNAs among lncRNAs overexpressed in CRPC tissues (Type_A and Type_B) by using RNA-seq data in prostate cancer cells. (e) Identification of AR-regulated lncRNAs in 22Rv1 cells. We used RNA-seq data to obtain AR-repressed (Fold > 1.5 by siAR) or AR-induced (Fold < 0.8 by siAR) lncRNAs.

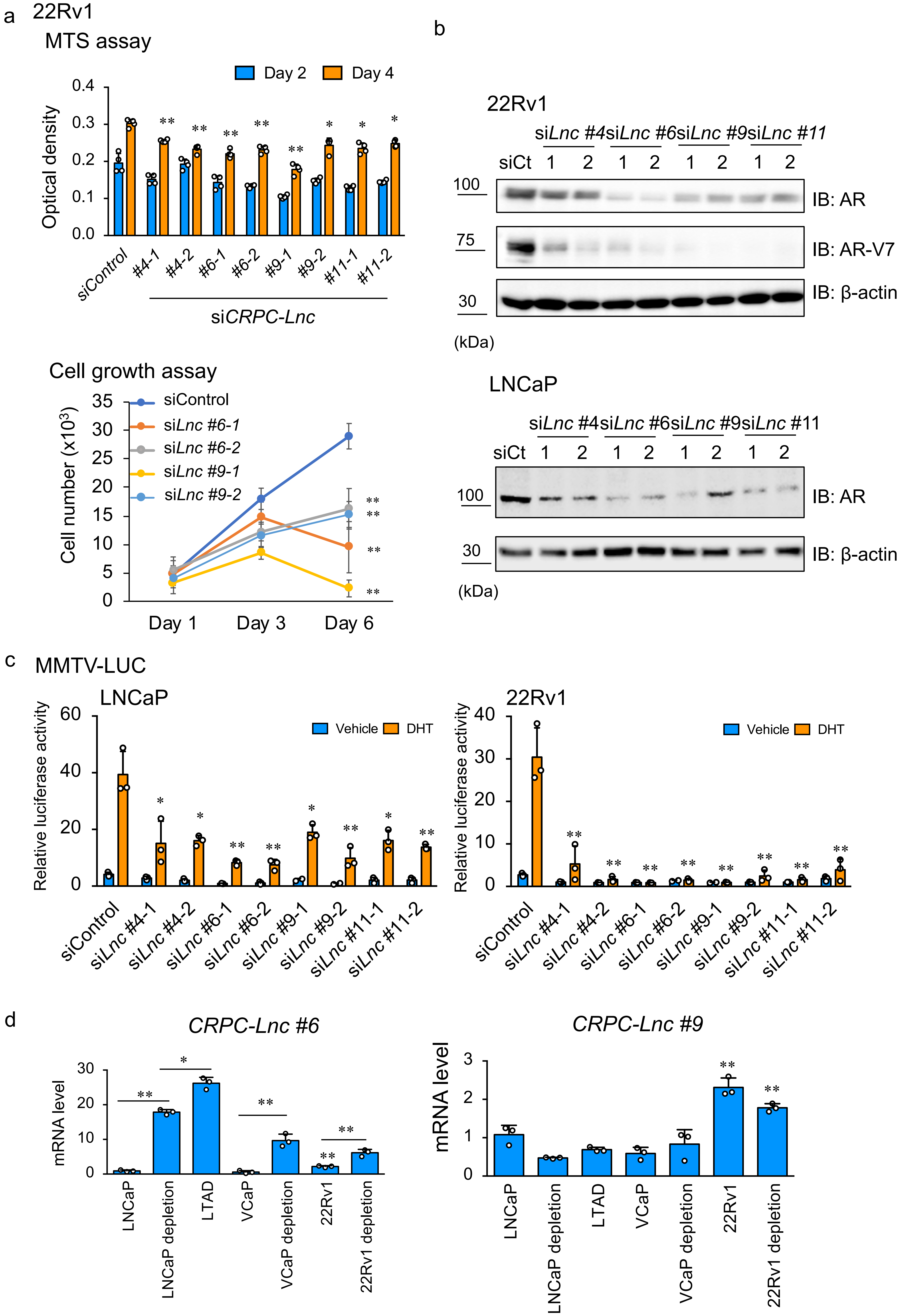
a
22Rv1
MTS assay
Day 2
Day 4
Optical density
0.4
0.3
0.2
0.1
0.0
siControl
#4-1
#4-2
#6-1
#6-2
#9-1
#9-2
#11-1
#11-2
siCRPC-Lnc
Cell growth assay
Cell number (x10³)
35
30
25
20
15
10
5
0
siControl
siLnc #6-1
siLnc #6-2
siLnc #9-1
siLnc #9-2
Day 1
Day 3
Day 6
b
22Rv1
siLnc #4 siLnc #6 siLnc #9 siLnc #11
siCt 1 2 1 2 1 2 1 2
100
75
30
IB: AR
IB: AR-V7
IB: β-actin
(kDa)
LNCaP
siLnc #4 siLnc #6 siLnc #9 siLnc #11
siCt 1 2 1 2 1 2 1 2
100
30
IB: AR
IB: β-actin
(kDa)
c
MMTV-LUC
LNCaP
Relative luciferase activity
60
40
20
0
Vehicle
DHT
siControl
siLnc #4-1
siLnc #4-2
siLnc #6-1
siLnc #6-2
siLnc #9-1
siLnc #9-2
siLnc #11-1
siLnc #11-2
22Rv1
Relative luciferase activity
40
30
20
10
0
Vehicle
DHT
siControl
siLnc #4-1
siLnc #4-2
siLnc #6-1
siLnc #6-2
siLnc #9-1
siLnc #9-2
siLnc #11-1
siLnc #11-2
d
CRPC-Lnc #6
mRNA level
30
20
10
0
LNCaP
LNCaP depletion
LTAD
VCaP
VCaP depletion
22Rv1
22Rv1 depletion
CRPC-Lnc #9
mRNA level
3
2
1
0
LNCaP
LNCaP depletion
LTAD
VCaP
VCaP depletion
22Rv1
22Rv1 depletion

Supplementary Figure 4. Additional data about the role of *CRPC-Lncs* in cell growth and AR activity.

(a) Cell proliferation was attenuated by knockdown of *CRPC-Lncs* (*#4, #6, #9*, and *#11*) using two siRNAs. MTS assay (upper) and cell counting (lower) were performed to assess cell growth ability in 22Rv1 cells (N = 4). *Lnc*: *CRPC-Lnc*.

(b) AR expression is inhibited by silencing of *CRPC-Lncs* (*#4, #6, #9*, and *#11*) using two siRNAs. Western blot analysis was performed in 22Rv1 and LNCaP cells. IB: immunoblot. Values represent the mean ± S.D. ∗P < 0.05, ∗∗P < 0.01.

(c) AR activity was repressed by knockdown of *CRPC-Lncs* (*#4, #6, #9*, and *#11*) using two siRNAs. *MMTV*-LUC was transfected after 48 h treatment of siRNAs. After 24 h incubation, cells were treated with 10 nM DHT or vehicle. Luciferase assay was performed for evaluation of AR activity (N = 3). *Lnc*: *CRPC-Lnc*. Values represent the mean ± S.D. ∗P < 0.05, ∗∗P < 0.01.

(d) *CRPC-Lncs* were distinctly affected by AR overexpression and androgen-deprivation. For hormone deprivation treatment, LNCaP, VCaP, and 22Rv1 cells were cultured in phenol-red free medium with 10 % charcoal dextran stripped FBS for one week. Expression levels of *CRPC-Lnc #6* and *#9* were determined by qRT-PCR analysis (N = 3). Values represent the mean ± S.D. ∗P < 0.05, ∗∗P < 0.01. LTAD: long-term androgen deprivation cells.

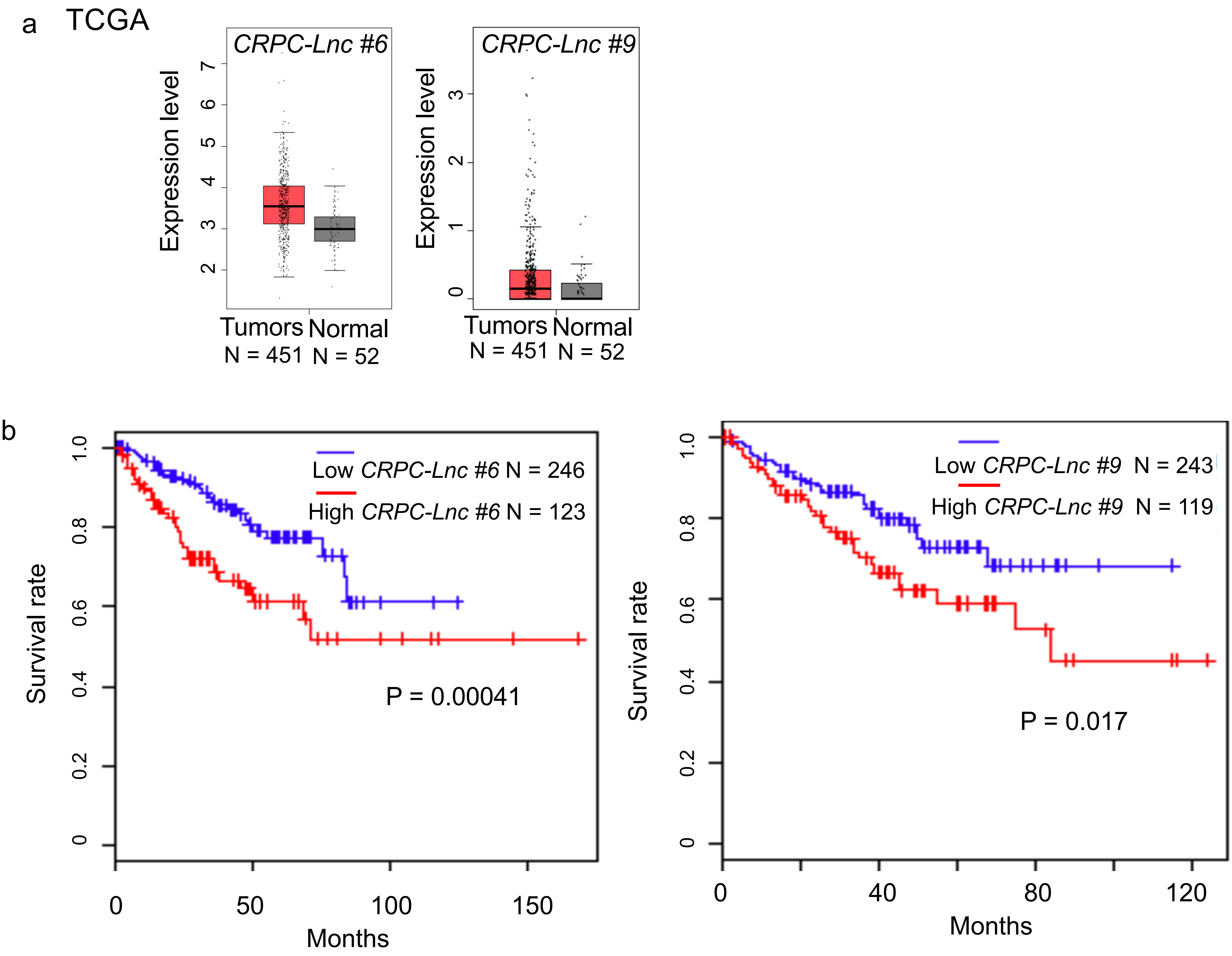

Supplementary Figure 5. High expression levels of *CRPC-Lncs* are correlated with poor prognosis of prostate cancer patients.

(a) Expression level of *CRPC-Lnc #6* and *#9* in prostate tumors and normal prostate tissues in the Cancer Genome Atlas (TCGA) cohort. We analyzed the expression levels of *CRPC-Lncs* by using Gene Expression interactive Analysis (GEPIA) (http://gepia.cancer-pku.cn/index.html).

(b) High expression levels of *CRPC-Lnc #6* and *#9* in prostate tumors are correlated with poor prognosis. We analyzed the disease-free survival of patients by using GEPIA. We set the cut off at 75% for high expression and 50 % for low of 451 tumors. P-value was calculated by log rank test.

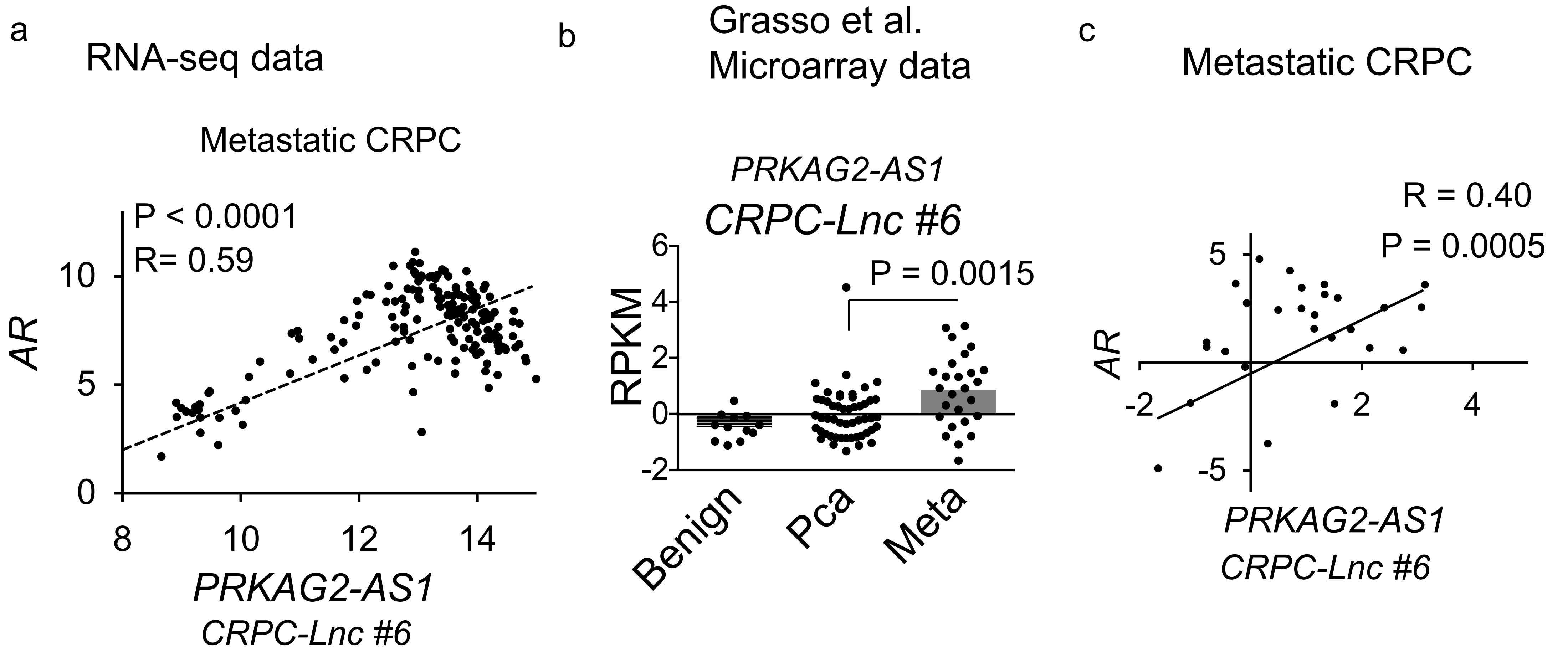

Supplementary Figure 6. The expression level of *CRPC-Lnc #6* is associated with *AR* mRNA.

(a) We analyzed the expression levels of *CRPC-Lnc* #6 (*PRKAG2-AS1*) and *AR* by using cbioportal RNA-seq data (Kumar et al.[1]). Spearman correlation analysis was performed to determine the correlation.

(b) *CRPC-Lnc #6* is upregulated in metastatic CRPC tissues compared with Pca and benign. We used publicly available microarray data (Grasso et al.[2]) to analyze the expression levels of *CRPC-Lnc #6* (*PRKAG2-AS1*). Meta: metastatic CRPC. Mann-Whitney U test was used to determine the P-value. (c) We analyzed the correlation of AR with *CRPC-Lnc #6* in metastatic CRPC tissues (N = 27) in microarray data (Grasso et al.[2]). Spearman correlation analysis was performed to determine the correlation.

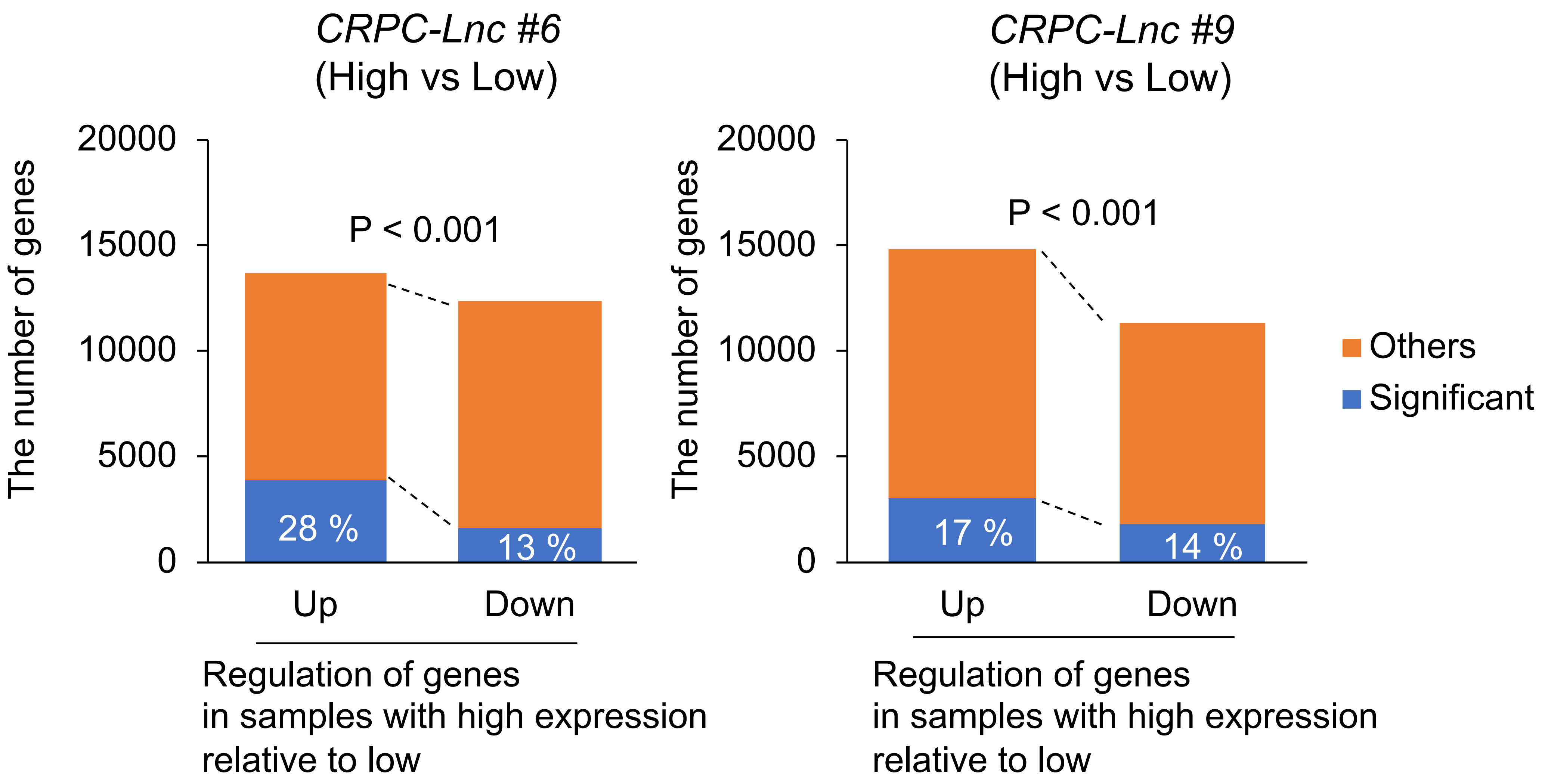

Supplementary Figure 7. The general effect of *CRPC-Lncs* on global gene expression.
The expression levels of *CRPC-Lnc #6* and *#9* in 154 prostate cancer tissues were analyzed using exon array data publicly available (Taylor et al.[3]). We then divided samples to two groups, “high expression” and “low expression” of *CRPC-Lncs* and compared the global expression profile between them. The rates of significantly upregulated genes ($P < 0.01$, tow-sided t-test) among all upregulated genes in high expression of *CRPC-Lncs* were larger than downregulated genes ($P < 0.0001$, chi-square test).

Fig.5a

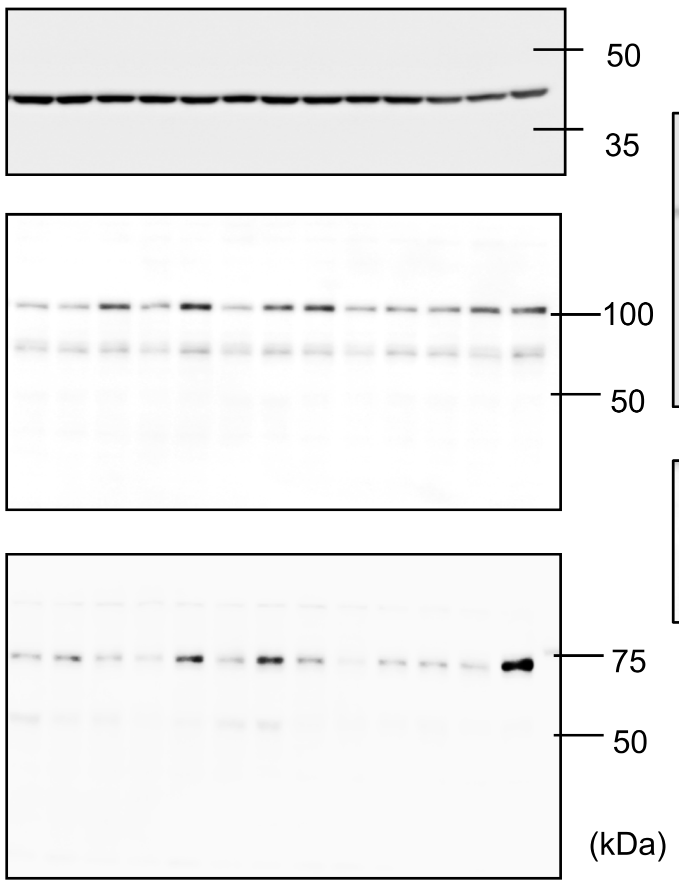

Fig.5b

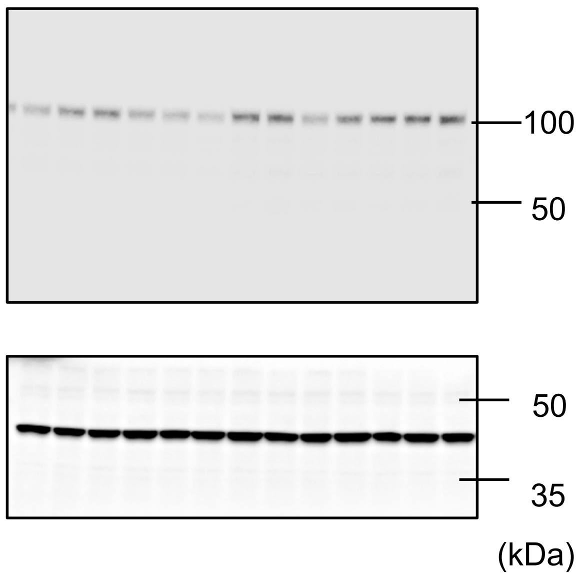

Fig.5g

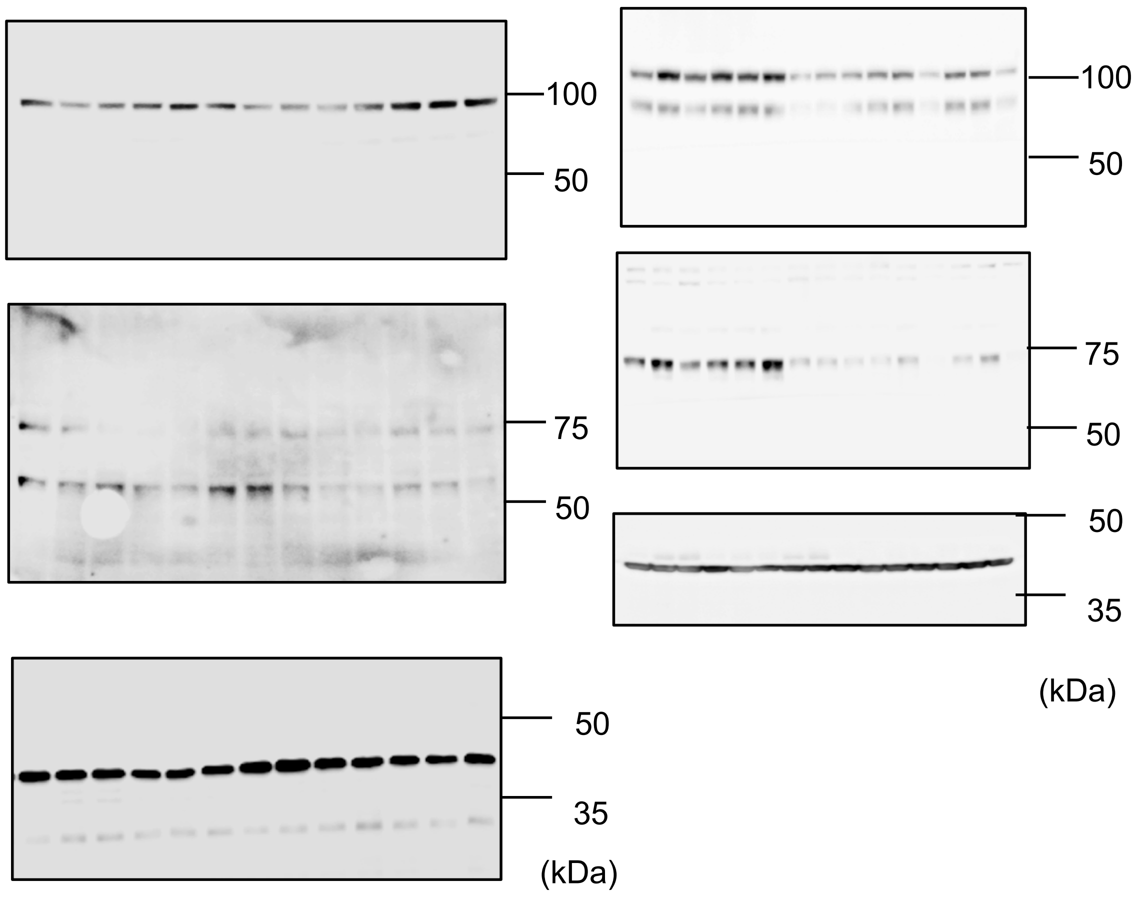

Fig.6b

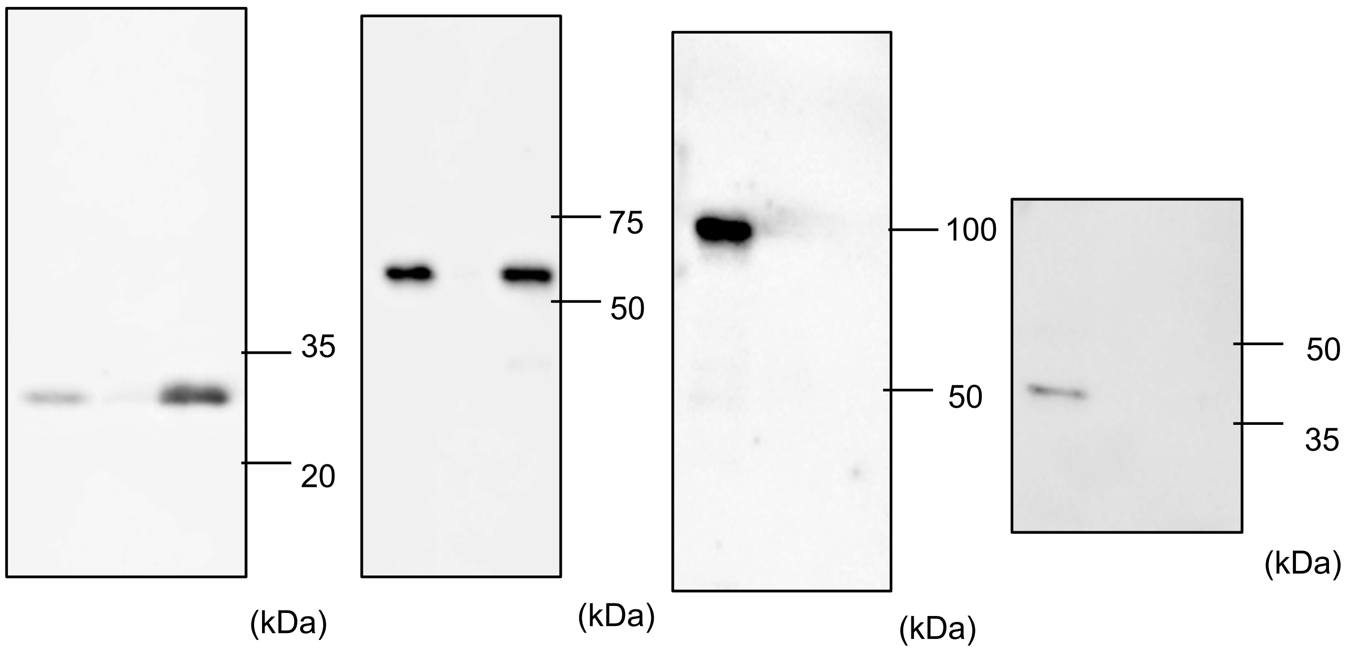

Fig.6g

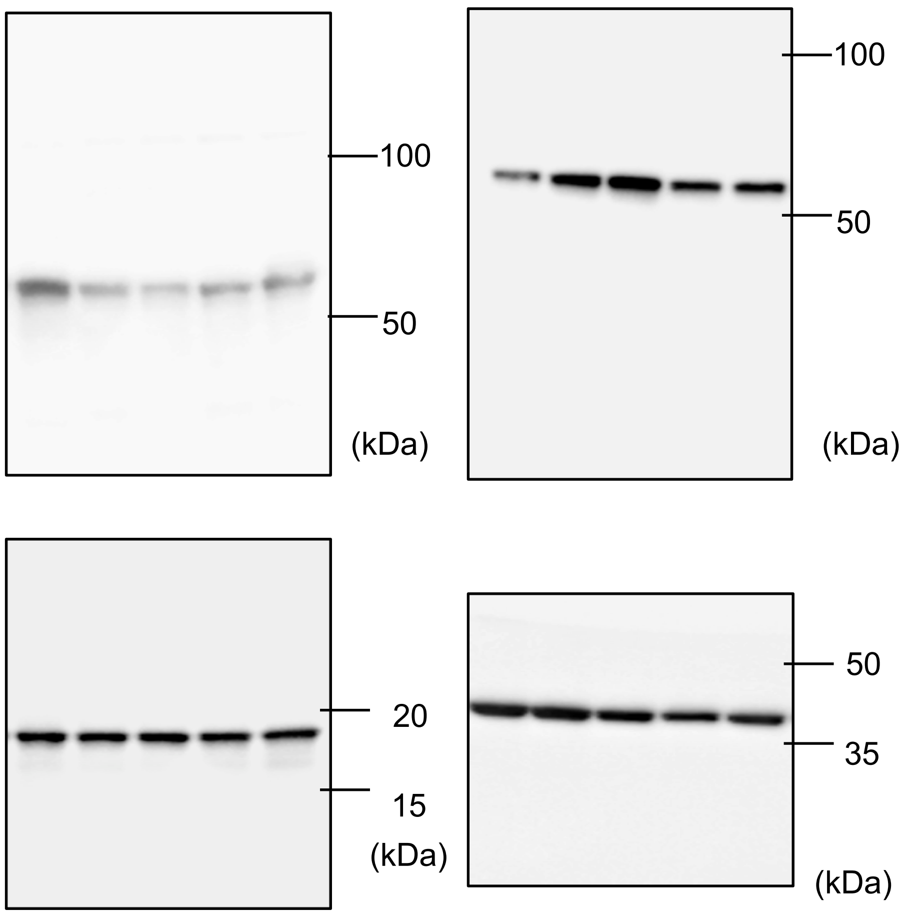

Fig.6f

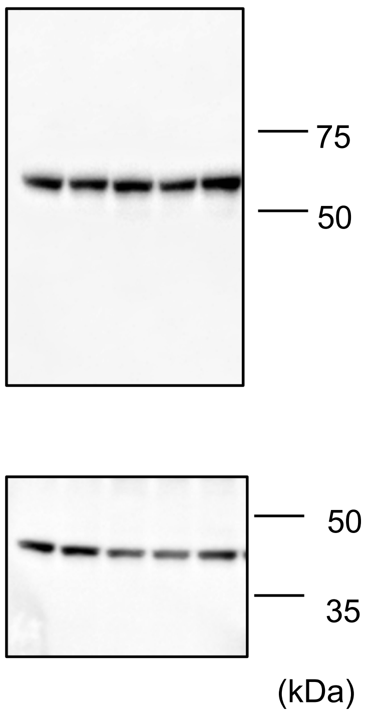

Fig.7c

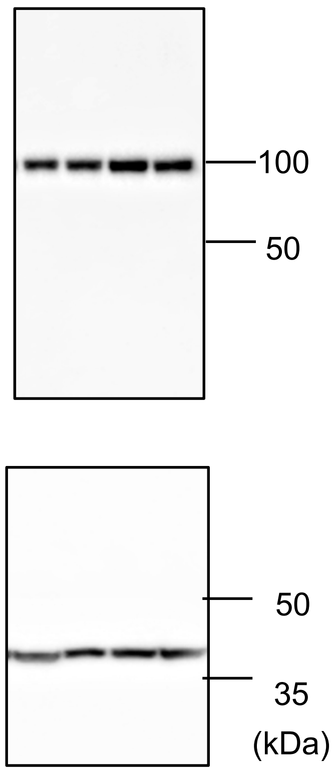

Fig.7f

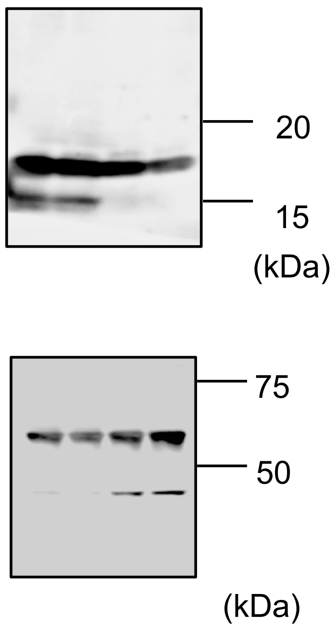

Supplementary Fig. S4

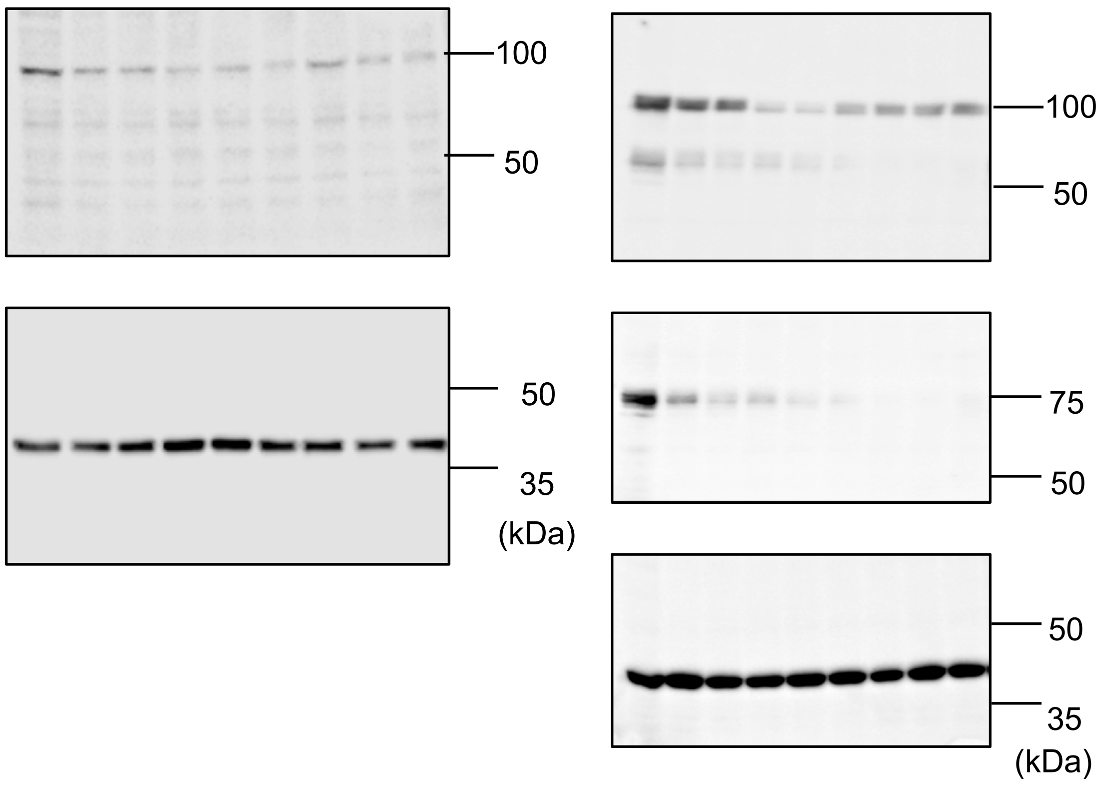

Supplementary Figure 8. Full scan images of immunoblotting in Figures.

Supplementary Table 1. Clinical characteristics of CRPC tissue samples used for RNA-seq analysis

	Case 1	Case 2	Case 3	Case 4	Case 5
Age	84	86	65	80	68
Methods to obtain tissues	TURP	TURP	Biopsy	Autopsy	TURP
PSA nadir	0.01	0.11	0.54	15.97	< 0.01
Time to PSA failure (days)	1761	840	3317	161	162
Serum PSA	0.01	15.05	8.01	25.13	5.9
First-treatment	MAB	MAB	Brachytherapy	MAB	RARP
Surgical castration	1	0	1	1	0
LHRH agonist	0	1	1	0	1
Casodex	1	1	0	1	1
Enzaltamide	1	0	0	0	1
Abiraterone	0	0	0	0	1
Radiotherapy	1	0	0	1	1

TURP: transurethral resection of the prostate, MAB: maximal androgen blockade, RARP: robot-assisted laparoscopic radical prostatectomy, 0: no, 1: yes We obtained two samples (lymph node and liver metastases) from Case 4.

Supplementary Table 2. Sequences of RNA FISH probes

CRPC-Lnc #6

Probe number	Sequence
1	ttccagattccccacttg
2	gcacgaacgggcttactg
2	ctccgagctggtttattctg
3	actgcgcaaaccttgatgaa
5	ttccaggctggcttcatt
6	ctctgtctcgagactggg
7	taaacccagacagggtcc
8	aggatggctgcttggttg
6	caggagaagacccagaagtc
7	aaatcctctagcaagaagcc
8	caatacagtcttgctgggtc
9	tatcttgagcattcagtggg
10	tccccttttctacttctaac
11	agtggggacagaggctaaat
20	gacaattgggaataattt
21	gctaaacagatgacacaa
22	gacaaggattaaaactgt
12	ccctggcaaatgatcatttt
13	ccagttctcatcaaataggg
17	gccccattttcctttaaa
14	agacgtctcttcacaccaac
17	cacccagtggaatgaggg
18	atcgcggttttctccttc
29	acactgtccataaagaaaac
30	gtacacattataacaattgc
31	gtatacagatcaatcaacaa
34	aatgtaggaagcactaaagt
35	gcaggcaatacagtaaaatg
*AR*_intron 3	
1	gctacttgagatgattcttttg
2	tatctgagtctcatttgatgtg
3	ttgagtgtataagaaccctcaa

4	tttgcatccaaatgttatgagg
5	caggctgcttaataatggagaa
6	actttccagctaaattactagt
7	tatggcataccaagttacttag
8	actttttcgtttgaggtaatca
9	ggaacgtggagataagttcatt
10	ctgtgcattgaatgtgtaagtt
11	agtctactccttaaggaataga
12	tatcaaagcactcaagtctcac
13	taagtaggtgtgaatggaggta
14	ttcagaaacaacaacagctgct
15	tttatcctaactatggactgtg
16	ttggggaggaataggaggaaag
17	actcaagagttgcaaaacgcag
18	tgctagacgaatctgtgaagta
19	cctctcaactagataattcaca
20	atgccatgaatagtaattcagc
21	tgcttgaagtctaaactaccta
22	ctttctacaatttaaaccctca
23	atgctagaaatttctctctagc
24	gtaactacaaatcctaggtgtt
25	aaccagtacttcttcatatacg
26	aaggaaactggcttttggtatg
27	ctgcttttgatcattaatgcat
28	tttagtggcattagtagctaat
29	tacctgcagacattagataact
30	caaaatgttggtcagacagtgt
31	tcaatcattggatggtttctac
32	ccacgttgttaattctttatga
33	aggagaatggaagaaatcccag
34	aaaagaagggctgaagggtacg
35	agtggaaatttcaagggttgtt
36	cccttaagaactacagttagag
37	attttggagcactcacagtaaa
38	tttggaaaatattgcctatggc
39	ttcttaacaacgtgatcccaaa

40	gaagaatggaacctgattccaa
41	ccctcaacaatcaatttttact
42	cggtgcttacatgtttttaatt
43	aagcattattactcagactgga
44	cactcagagaccaagaaagact
45	ccacttgtaagacagaagtgag
46	cataggaagcagcatgtaaagt
47	aatcagatgcctaagacagatc
48	tgttgactgtagcagtctaaat

Supplementary Table 3. Primer sequences for qRT-PCR

Primer	Sequence
#1:*NR_039988*	F: CAGTGAGCAGAACCAGTCCA
	R: TAGAAGCAGGGCTTTCCTCA
#2:*LINC00665*	F: TCCTGACCTCTGACCCGTAT
	R: ATGGTAGTCGATCCGCTGTC
#3:*DANCR*	F: TGCAGCTGCCTCAGTTCTTA
	R: ACCTGCTACACTGCCCTGTT
#4:*FAM83H-AS1*	F: GCGACAGGAGAATGGACAGT
	R: GGAGGTAGCAGTGAGCCAAG
#5:*LOC101927746*	F: TTCACACTTGCAGCAGATCC
	R: CTTTTTCACGTTTCCCGTGT
#6:*PRKAG2-AS1*	F: GACCCTGTCTGGGTTTAGCA
	R: ACTACCCCCTCCCCTTTTCT
#7:*RPARP-AS1*	F: GCCCGGCCTATTTACTTTTC
	R: TTTTTGGCTTCTCCCATGAC
#8:*FOXP4-AS1*	F: AAAACCACGTGCACCAAACT
	R: AGCAGACCTGGAGCTGTCAT
#9:*HOXC-AS1*	F: CATGTCCACCCATAAGCAGA
	R: GTGTCGCAGAGATGGAGTTG
#10:*SNHG19*	F: GCACCACTTTGTCCCTTAGC
	R: ATAGACACAGCGCCCACCT
#11:*ELFN1-AS1*	F: GCCTCAGCCACAATCGTAAT
	R: CAGGTTCTTCAGCCAGGAAG
#12:*ERVK3-1*	F: TGGCCATGCTAGCTGTAGTG
	R: GAGTTGGTCACGACCCTCAT
RP11-539I5.1	F: GCAGCAGCTACCACACAAAG
	R: TTGGCTAACAGGCCCATAAC
TPPPP	F: ACTGGACAGGGTCAAGGATG
	R: GCTCAGAAGGATGCCTGAAC
ELF3-AS1	F: AGAATCCACACGGAATCCAC
	R: CTGACTGAACCCAAGCCATT
RORB-AS1	F: CAGCACTGCTCTTCTGCTTG
	R: GACAGGAGCAGTTTGGAAGC
FOLH1-intron	F: AGGGAAGAGGTCAGAGCACA
	R: GCTTCAGCTTCGTAGCCACT

LOC100294145	F: GGTCTGTACCTGAGCCTGGA
	R: CATGCTGAGGTTCCCAATTT
HOXC-intron	F: ATCTGTTTCTCCCCCTGCTT
	R: AGGTCTTGTCCTGGCTCAGA
ARLNC1	F: ACCTTGTCCACTGGAACTCG
	R: ATCCGCAAAGGCACTATTTC
RN7SL1	F: GGAGTTCTGGGCTGTAGTGC
	R: ATCAGCACGGGAGTTTTGAC
