# Supplementary material for: Identification of long non-coding RNAs in advanced prostate cancer associated with androgen receptor splicing factors
Source: Commun Biol. 2020 Jul 23;3:393. doi: 10.1038/s42003-020-01120-y (PMC7378231; doi:10.1038/s42003-020-01120-y)
Supplement: Supplementary file 2 — Description of Additional Supplementary Files [file 42003_2020_1120_MOESM2_ESM.pdf]

Description of Additional Supplementary Files

File Name: **Supplementary Data 1**
Description: Summary of genes (RefSeq, NONCODE, GENCODE) identified by RNA-seq analysis to be upregulated in CRPC

File Name: **Supplementary Data 2**
Description: The source data for the graphs in the main figures and supplementary figures
