# Supplementary material for: Identification of long non-coding RNAs in advanced prostate cancer associated with androgen receptor splicing factors
Source: Commun Biol. 2020 Jul 23;3:393. doi: 10.1038/s42003-020-01120-y (PMC7378231; doi:10.1038/s42003-020-01120-y)
Supplement: Supplementary file 5 — Reporting Summary [file 42003_2020_1120_MOESM5_ESM.pdf]

nature research

Corresponding author(s): Satoshi Inoue

Last updated by author(s): May 14, 2020

Reporting Summary

Nature Research wishes to improve the reproducibility of the work that we publish. This form provides structure for consistency and transparency in reporting. For further information on Nature Research policies, see our Editorial Policies and the Editorial Policy Checklist.

Statistics

For all statistical analyses, confirm that the following items are present in the figure legend, table legend, main text, or Methods section.

n/a	Confirmed	
☐	☒	The exact sample size (*n*) for each experimental group/condition, given as a discrete number and unit of measurement
☐	☒	A statement on whether measurements were taken from distinct samples or whether the same sample was measured repeatedly
☐	☒	The statistical test(s) used AND whether they are one- or two-sided<br>*Only common tests should be described solely by name; describe more complex techniques in the Methods section.*
☒	☐	A description of all covariates tested
☒	☐	A description of any assumptions or corrections, such as tests of normality and adjustment for multiple comparisons
☐	☒	A full description of the statistical parameters including central tendency (e.g. means) or other basic estimates (e.g. regression coefficient) AND variation (e.g. standard deviation) or associated estimates of uncertainty (e.g. confidence intervals)
☐	☒	For null hypothesis testing, the test statistic (e.g. F, t, r) with confidence intervals, effect sizes, degrees of freedom and P value noted<br>*Give P values as exact values whenever suitable.*
☒	☐	For Bayesian analysis, information on the choice of priors and Markov chain Monte Carlo settings
☒	☐	For hierarchical and complex designs, identification of the appropriate level for tests and full reporting of outcomes
☐	☒	Estimates of effect sizes (e.g. Cohen's *d*, Pearson's *r*), indicating how they were calculated

Our web collection on statistics for biologists contains articles on many of the points above.

Software and code

Policy information about availability of computer code

Data collection	Bowtie 2 v. 2.2.6 and Tophat 2.1.0 were used for mapping sequenced tags to human genome hg19.
Data analysis	DAVID 6.7 (Pathway analysis), GraphPad Prism ver. 6.0 (chi-square test, Spearman correlation test, two way ANOVA), microsoft Excel (two sided t-test)

For manuscripts utilizing custom algorithms or software that are central to the research but not yet described in published literature, software must be made available to editors and reviewers. We strongly encourage code deposition in a community repository (e.g. GitHub). See the Nature Research guidelines for submitting code & software for further information.

Data

Policy information about availability of data

All manuscripts must include a data availability statement. This statement should provide the following information, where applicable:

- Accession codes, unique identifiers, or web links for publicly available datasets
- A list of figures that have associated raw data
- A description of any restrictions on data availability

All RNA-seq data have been deposited in the Japanese Genotype-phenotype Archive (JGA) under accession code JGAS0000000019846. The Gene expression omnibus (GEO) accession numbers for sequence data (RNA-seq and ChIP-seq) used in this study are GSE141806, GSE123565, GSE94577, and GSE82225.

Field-specific reporting

Please select the one below that is the best fit for your research. If you are not sure, read the appropriate sections before making your selection.

☒ Life sciences ☐ Behavioural & social sciences ☐ Ecological, evolutionary & environmental sciences

For a reference copy of the document with all sections, see nature.com/documents/nr-reporting-summary-flat.pdf

Life sciences study design

All studies must disclose on these points even when the disclosure is negative.

Sample size	Sample sizes were equal or greater than the recommended minimum sample size in the past publications.
Data exclusions	We excluded samples if the obtained values were more than twice the s.d. of the mean.
Replication	qRT-PCR was performed to validate the data of RNA-seq. Other experiments were reproduced at least twice.
Randomization	In the experiment of xenograft, the mice were randomly divided into four groups.
Blinding	No blind experiments

Reporting for specific materials, systems and methods

We require information from authors about some types of materials, experimental systems and methods used in many studies. Here, indicate whether each material, system or method listed is relevant to your study. If you are not sure if a list item applies to your research, read the appropriate section before selecting a response.

Materials & experimental systems

n/a	Involved in the study	
☐	☒	Antibodies
☐	☒	Eukaryotic cell lines
☒	☐	Palaeontology and archaeology
☐	☒	Animals and other organisms
☐	☒	Human research participants
☒	☐	Clinical data
☒	☐	Dual use research of concern

Methods

n/a	Involved in the study	
☒	☐	ChIP-seq
☒	☐	Flow cytometry
☒	☐	MRI-based neuroimaging

Antibodies

Antibodies used	Rabbit polyclonal anti-AR (ab108341), and anti-AR-V7 (ab198394) antibodies were purchased from Abcam (Cambridge, UK). The rabbit polyclonal anti-H3 (4499) antibody was purchased from Cell Signaling Technology (Danvers, MA). The mouse monoclonal anti-β-actin (A2228) antibody were obtained from Sigma. The mouse monoclonal anti-U2AF2 (MC3), anti-hnRNAPA1 (4B10), and a goat polyclonal anti-GAPDH (V18) antibodies were purchased from Santa Cruz Biotechnology (Dallas, TX).
Validation	All antibodies were validated in manufacturer's web pages or our past studies.

Eukaryotic cell lines

Policy information about cell lines

Cell line source(s)	The cell lines used in the present study were obtained from ATCC.
Authentication	Identities of the cells were confirmed by short tandem repeat (STR) analyses in 2019 and 2015 (BEX co. Ltd., Tokyo, Japan).
Mycoplasma contamination	We routinely checked for Mycoplasma contamination using a PCR-based kit, Mycoplasma detection kit (Jena Bioscience, Jena, Germany).
Commonly misidentified lines (See ICLAC register)	None

Animals and other organisms

Policy information about studies involving animals; ARRIVE guidelines recommended for reporting animal research

Laboratory animals	5-week-old male BALB/c nude mice (CLEA Japan)
Wild animals	None
Field-collected samples	None
Ethics oversight	Animal care was in accordance with the Tokyo Metropolitan Institute of Gerontology animal experiment guidelines. The ethics committee of animal experiments at the Tokyo Metropolitan Institute of Gerontology approved our study protocol.

Note that full information on the approval of the study protocol must also be provided in the manuscript.

Human research participants

Policy information about studies involving human research participants

Population characteristics	The age of prostate cancer patients ranged from 57-75 years and the pretreatment serum PSA levels were 4.1-32 ng/mL. A total of six CRPC fresh-frozen tumors were obtained from five patients, who underwent hormone-, chemo-, and radiotherapy.
Recruitment	We obtained prostate cancer samples from surgeries, biopsies, and pathological anatomies. Written informed consent was obtained from each patient before treatment.
Ethics oversight	The study was approved by the Human Genome, Gene Analysis Research Ethics Committee of the Jichi Medical University (No. 18-15), Tokyo Metropolitan Institute of Gerontology (No. 1183), and University of Tokyo (G10044).

Note that full information on the approval of the study protocol must also be provided in the manuscript.
